# Three years of wastewater surveillance for new psychoactive substances from 16 countries

**DOI:** 10.1016/j.wroa.2023.100179

**Published:** 2023-04-06

**Authors:** Richard Bade, Nikolaos Rousis, Sangeet Adhikari, Christine Baduel, Lubertus Bijlsma, Erasmia Bizani, Tim Boogaerts, Daniel A. Burgard, Sara Castiglioni, Andrew Chappell, Adrian Covaci, Erin M. Driver, Fernando Fabriz Sodre, Despo Fatta-Kassinos, Aikaterini Galani, Cobus Gerber, Emma Gracia-Lor, Elisa Gracia-Marín, Rolf U. Halden, Ester Heath, Felix Hernandez, Emma Jaunay, Foon Yin Lai, Heon-Jun Lee, Maria Laimou-Geraniou, Jeong-Eun Oh, Kristin Olafsdottir, Kaitlyn Phung, Marco Pineda Castro, Magda Psichoudaki, Xueting Shao, Noelia Salgueiro-Gonzalez, Rafael Silva Feitosa, Cezar Silvino Gomes, Bikram Subedi, Arndís Sue Ching Löve, Nikolaos Thomaidis, Diana Tran, Alexander van Nuijs, Taja Verovšek, Degao Wang, Jason M. White, Viviane Yargeau, Ettore Zuccato, Jochen F. Mueller

**Affiliations:** a Queensland Alliance for Environmental Health Sciences (QAEHS), The University of Queensland, 20 Cornwall Street, Woolloongabba, QLD 4102, Australia; b School of Sustainable Engineering and Built Environment, Arizona State University, Tempe, AZ, 85281, United States; c Biodesign Center for Environmental Health Engineering, Biodesign Institute, Arizona State University, 1001 S. McAllister Ave., Tempe, AZ 85281, United States; d Université Grenoble Alpes, CNRS, IRD, Grenoble INP, IGE, Grenoble, France; e Environmental and Public Health Analytical Chemistry, Research Institute for Pesticides and Water, University Jaume I, Avda, Sos Baynat s/n, E-12071 Castellón, Spain; f Laboratory of Analytical Chemistry, Department of Chemistry, National and Kapodistrian University of Athens, Panepistimiopolis Zografou, 15771 Athens, Greece; g Toxicological Centre, Department of Pharmaceutical Sciences, University of Antwerp, 2610 Wilrijk, Belgium; h Department of Chemistry and Biochemistry, University of Puget Sound, Tacoma, WA 98416, United States; i Istituto di Ricerche Farmacologiche Mario Negri IRCCS, Department of Environmental Health Sciences, Via Mario Negri 2, 20156, Milan, Italy; j Institute of Environmental Science and Research Limited (ESR), Christchurch Science Centre: 27 Creyke Road, Ilam, Christchurch 8041, New Zealand; k AquaVitas, LLC, Scottsdale, Arizona, 85251, United States; l Institute of Chemistry, University of Brasília, Brasília, DF, 70910-000, Brazil; m Nireas-International Water Research Centre and Department of Civil and Environmental Engineering, University of Cyprus, P.O. Box 20537, 1678, Nicosia, Cyprus; n Health and Biomedical Innovation, UniSA: Clinical and Health Sciences, University of South Australia, Adelaide 5001, South Australia, Australia; o Department of Analytical Chemistry, Faculty of Chemistry, Complutense University of Madrid, Avenida Complutense s/n, 28040 Madrid, Spain; p OneWaterOneHealth, Arizona State University Foundation, 1001 S. McAllister Avenue, Tempe, AZ 85287-8101, United States; q Jožef Stefan Institute and International Postgraduate School, Jamova 39, 1000 Ljubljana, Slovenia; r Department of Aquatic Sciences and Assessment, Swedish University of Agricultural Sciences (SLU), SE-75007 Uppsala, Sweden; s Department of Civil and Environmental Engineering, Pusan National University, Jangjeon-dong, Geumjeong-gu, Busan 46241, Republic of Korea; t University of Iceland, Department of Pharmacology and Toxicology, Hofsvallagata 53, 107 Reykjavik, Iceland; u Department of Chemical Engineering, McGill University, Montreal, QC, Abbreviation:; v College of Environmental Science and Engineering, Dalian Maritime University, No. 1 Linghai Road, Dalian, 116026, P. R. China; w Laboratory of Forensic Chemistry, Brazilian Federal Police, PB, Brazil; x Department of Chemistry, Murray State University, Murray, Kentucky 42071-3300, United States

**Keywords:** Illicit drugs, Public health, Monitoring, 3-methylmethcathinone, Wastewater analysis

## Abstract

The proliferation of new psychoactive substances (NPS) over recent years has made their surveillance complex. The analysis of raw municipal influent wastewater can allow a broader insight into community consumption patterns of NPS. This study examines data from an international wastewater surveillance program that collected and analysed influent wastewater samples from up to 47 sites in 16 countries between 2019 and 2022. Influent wastewater samples were collected over the New Year period and analysed using validated liquid chromatography – mass spectrometry methods. Over the three years, a total of 18 NPS were found in at least one site. Synthetic cathinones were the most found class followed by phenethylamines and designer benzodiazepines. Furthermore, two ketamine analogues, one plant based NPS (mitragynine) and methiopropamine were also quantified across the three years. This work demonstrates that NPS are used across different continents and countries with the use of some more evident in particular regions. For example, mitragynine has highest mass loads in sites in the United States, while eutylone and 3-methylmethcathinone increased considerably in New Zealand and in several European countries, respectively. Moreover, 2F-deschloroketamine, an analogue of ketamine, has emerged more recently and could be quantified in several sites, including one in China, where it is considered as one of the drugs of most concern. Finally, some NPS were detected in specific regions during the initial sampling campaigns and spread to additional sites by the third campaign. Hence, wastewater surveillance can provide an insight into temporal and spatial trends of NPS use.

## Highlights

• Up to 47 sites in 16 countries monitored over three years.
• Synthetic cathinones most commonly found class.
• Highest loads for mitragynine, particularly in sites from the United States.
• Some temporal and regional trends evident.

## Graphical abstract

**Figure:**
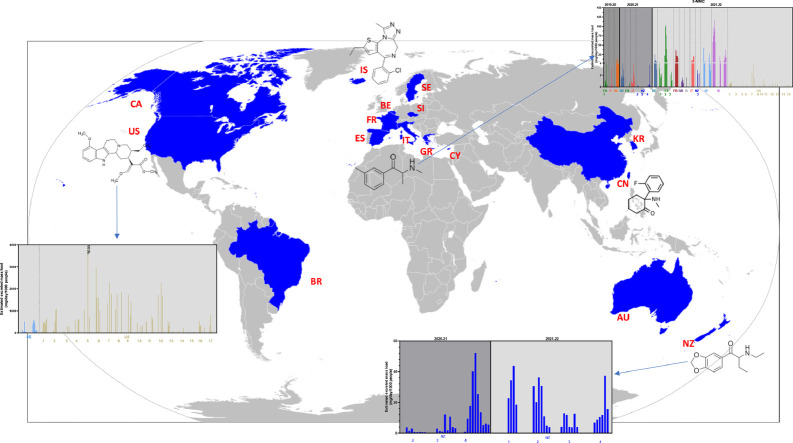

## 1 Introduction

New psychoactive substances (NPS) are compounds that have been designed to mimic the effect of conventional illicit drugs, while evading legal restrictions and are thus of international public health concern. From fentanyl-laced heroin (Jannetto et al., 2019) to counterfeit Xanax (Blakey et al., 2022) and adulterated MDMA (Krotulski et al., 2021; HighAlert, 2021), NPS may pose a risk to illicit users. The United Nations Office of Drugs and Crime (UNODC) runs an Early Warning Advisory (EWA) program on NPS, which is updated with information from international drug enforcement and intelligence agencies, health authorities and toxicologists. As of mid-2022, 136 countries and territories, covering all continents had reported more than 1100 NPS to the EWA.

The consumption of NPS is influenced by their specific effects, availability, price, potential undetectability in routine drug tests and their use as adulterants (Peacock et al., 2019; United Nations Office on Drugs and Crime 2022). It is imperative from a public health viewpoint to monitor the use of these drugs given our limited knowledge of their specific effects, their interactions with other drugs, and the harms that they cause. It is difficult for law enforcement to control the circulation of these compounds because they are generally manufactured in much smaller quantities than traditional illicit drugs such as cocaine and methamphetamine and many have a ‘grey’ legal status.

Information on the prevalence of NPS use is collected from forensic analyses, surveys, and pill testing as well as social media and dark web monitoring (Peacock et al., 2019; Pascoe et al., 2022; Barenholtz et al., 2021). Each of these serves as a complementary tool for identification, monitoring, surveillance, control and ultimately evaluation of public health impacts of NPS use. Each has its distinct advantages such as rapid identification of substances, early warning capabilities and outlining demographics and profiles of users. However, not all jurisdictions have access to these data sources and thus some communities could be misinformed. There is increased concern around music festivals, where NPS-adulterated drugs can be mistakenly consumed. This can increase the risk of overdoses, resulting in more emergency department presentations. Wastewater analysis can help to fill this gap and has been used in many countries to assess licit and illicit drug use (Gracia-Lor et al., 2017).

The global prevalence of NPS use remains unknown and data are limited to a few countries with appropriate resources (UNODC 2022; Khaled et al., 2016). The current work presents data from wastewater sampling across the New Year period in three consecutive years (2019–20, 2020–21 and 2021–22). The use of NPS typically increases during festivals such as those of the New Year period that are associated with parties and festivals. The number of countries (and sites) has increased from 8 (12) in the first iteration to 10 (25) and finally to 16 (47). Throughout this project, the number of targeted analytes changed to include the most relevant NPS, based on findings from the UNODC EWA on NPS, international forensic findings, and published scientific literature. The aims of this work are: i) to study international spatial trends in NPS use; ii) to examine if preferences for NPS drugs change from year to year; iii) to evaluate the impact of the COVID-19 pandemic on NPS use and iv) to establish whether data on NPS obtained from wastewater analysis is comparable to that from other data sources.

## 2 Results

Across the three years, the number of countries (and sites) investigated increased from 8 (12) to 10 (25) and finally 16 (47). Across all three campaigns, a total of 546 individual samples were analysed, between 115 (2019/20) and 287 (2021/22). A total of 18 NPS were found across the three sampling campaigns (Table 1, structures in Table S1), with yearly totals from nine (2019–20 and 2021–22) to ten (2020–21) individual NPS. Each year, the number of analytes included in the analytical method has changed (Table S2) based on information from the UNODC EWA, forensic agencies, peer reviewed publications and availability of reference analytical standards. For example, 3-methylmethcathinone (3-MMC), mephedrone and methylone were analysed across all years, while 2F-deschloroketamine (2F-DCK), mitragynine, clonazolam and etizolam were only included in the third campaign. 4-Methylethcathinone (4-MEC), methylenedioxypyrovalerone (MDPV), methiopropamine, methoxetamine and *para*-methoxyamphetamine (PMA) were not included in the 2021–22 campaign because there was limited identification in international forensic analyses and in the early warning systems of the UNODC and EMCDDA.

**Table 1:** Table of all NPS found over 3 years (Country with sites of the highest levels are highlighted in bold).

A variety of classes of NPS were found during this campaign. Synthetic cathinones were the most common (Table 1). Phenethylamines (4-fluoroamphetamine and PMA), designer benzodiazepines (clonazolam and etizolam), ketamine analogues (2F-DCK and methoxetamine) as well as the plant-based NPS mitragynine and methiopropamine were also quantified at least once across the three years.

### 2.1 Spatial trends

**Fig. 1:**
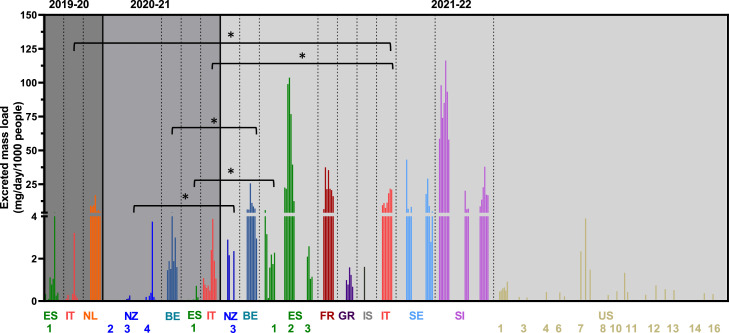
Estimated excreted mass loads of 3-MMC from all sites over the three-year sampling period. Note: 2019–20 and 2020–21 values are taken from previous publications (Bade et al., 2021; Bade et al., 2022). Sites are ordered by continent. Countries with multiple sites are numbered according to Table S3. Only sites where the compound was found are included in the figure. *: Statistically significant difference (*p* < 0.05) following independent t-tests (for sites with two years of data) or a one-way ANOVA, followed by a pairwise *t*-test with Bonferroni correction (for sites with three years of data). ES: Spain; IT: Italy; NL: the Netherlands’ BE: Belgium; NZ: New Zealand; FR: France; GR: Greece; IS: Iceland; SE: Sweden; SI: Slovenia; US: United States.

**Fig. 2:**
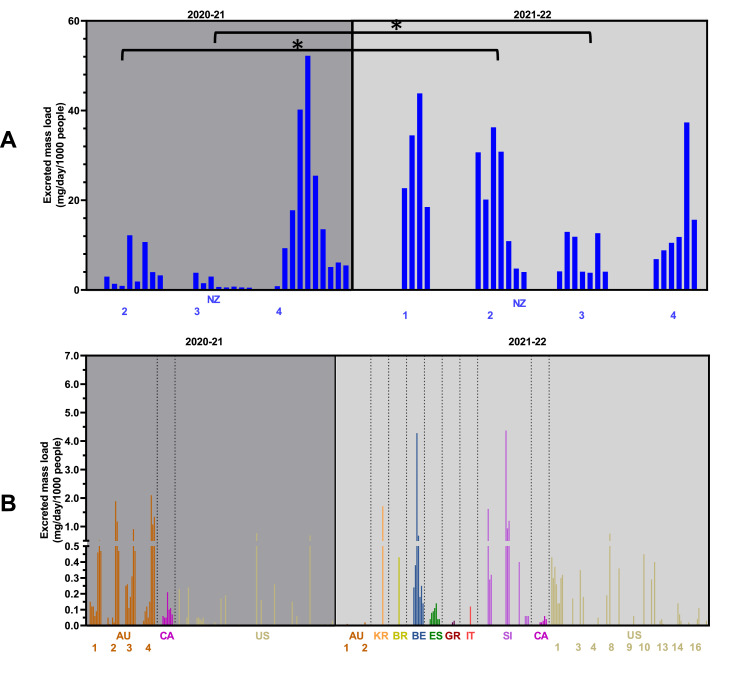
Estimated excreted mass loads of eutylone from all sites. Figure is separated to show the sites with highest levels (A) and the lower levels (B). Note: 2020–21 values are taken from a previous publications (Bade et al., 2022). Sites are ordered by continent. Countries with multiple sites are numbered according to Table S3. Only sites where the compound was found are included in the figure. *: Statistically significant difference (*p* < 0.05) following independent t-tests (for sites with two years of data). NZ: New Zealand; AU: Australia; CA: Canada; US: United States; BE: Belgium; BR: Brazil; ES: Spain; GR: Greece; IT: Italy; KR: Republic of Korea; SI: Slovenia.

Over the three years of these data, some spatial patterns emerged. The synthetic cathinone 3-MMC was found consistently in all years, primarily in Europe (Fig. 1). However, in 2020–21 and 2021–22, it was found in at least one site in New Zealand. In the latter sampling period, it was also found on selected days in the United States. Mephedrone and methylone were also found across the three campaigns. They were primarily located in Oceania and North America, with highest levels in New Zealand. Across the European sites, they were only found in one site in Spain in the 2021–22 collection (Table 1). Like mephedrone, eutylone was also primarily seen in sites in New Zealand (up to 55 mg/day/1000 people) in both 2020–21 and 2021–22 (Fig. 2A).

N-ethylpentylone, pentylone and ethylone had the lowest levels of synthetic cathinones found across the three sampling campaigns. N-ethylpentylone and pentylone had highest levels in the United States. Several compounds were only seen once: 4-fluoroamphetamine, 4-MEC and MDPV in the Netherlands, methiopropamine and methoxetamine in Australia and PMA in New Zealand (Table 1).

**Fig. 3:**
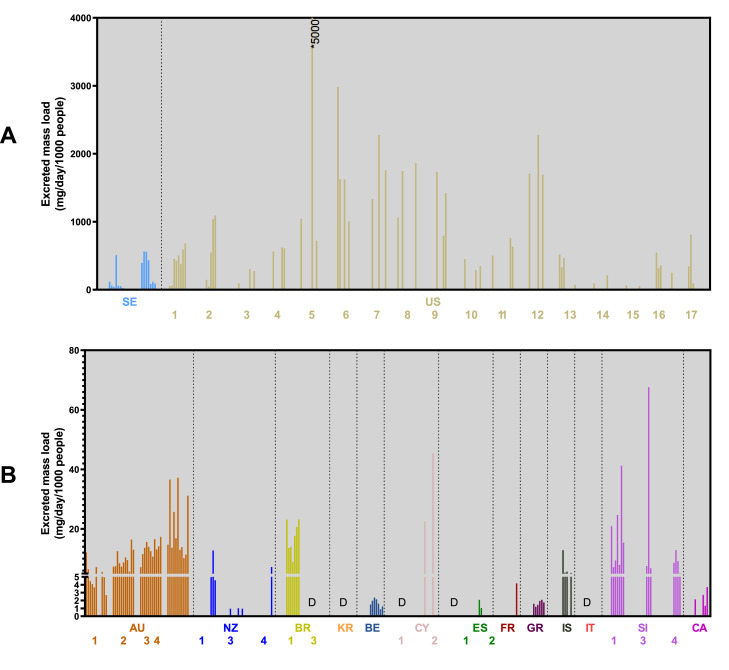
Estimated excreted mass loads of mitragynine from all sites from the 2021–22 collection. Sites are ordered by continent. Figure is separated to show the sites with highest levels (A) and the lower levels (B). *D* = detected at levels below LOQ. Countries with multiple sites are numbered according to Table S3. Only sites where the compound was found are included in the figure. AU: Australia; BE: Belgium; BR: Brazil; CA: Canada; CY: Cyprus; ES: Spain; FR: France; GR: Greece; IS: Iceland; IT: Italy; KR: Republic of Korea; NZ: New Zealand; SE: Sweden; SI: Slovenia US: United States.

Some substances were only quantified in the most recent surveillance campaign in 2021-22. Mitragynine (Fig. 3) was found at the highest levels in the United States, mostly between 1000 and 5000 mg/day/1000 people. Sites in Sweden had the next highest levels. Most other sites where the compound was found had levels below 50 mg/day/1000 people. There was generally no increase in levels over the New Year in any site except for specific sites in Slovenia and Sweden. N-ethylhexedrone was only quantifiable in two sites in Sweden and Spain, with particularly high levels in one site from Sweden on New Year's Day. The designer benzodiazepines clonazolam and etizolam were also found, with highest loads at a site in Iceland, while levels in wastewater at other sites were below the limit of quantification of our method.

**Fig. 4:**
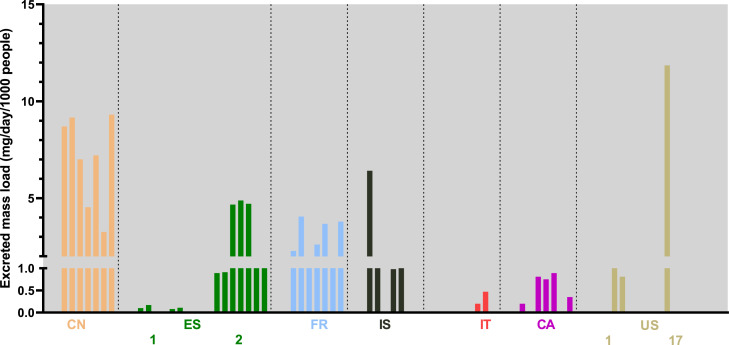
Estimated excreted mass loads of 2F-DCK from all sites from the 2021–22 collection. Sites are ordered by continent. Countries with multiple sites are numbered according to Table S3. Only sites where the compound was found are included in the figure. CA: Canada; CN: China; ES: Spain; FR: France; IS: Iceland; IT: Italy; US: United States.

2F-DCK was found in samples from nine sites across Canada, China, Spain, France, Iceland, Italy, and the United States (Fig. 4). Increases over New Year's Eve were evident in Canada and Spain (Table S4), while highest levels were seen across the entire sampling week in China. It was only quantifiable on certain days in Iceland, Italy, and the United States.

### 2.2 Temporal trends

For sites and compounds that were monitored over multiple years, statistical tests were performed to determine significance. 3-MMC was monitored across all three years and the measured excreted mass loads increased from 5 to 10 mg/day/1000 people in the first two sampling campaigns to more than 100 mg/day/1000 people in 2021–22. The highest levels were found in the 2021–22 sampling campaign at sites in Spain and Slovenia. In sites monitored over multiple years, there was also an increase (i.e. Belgium (BE), Spain (ES 1), New Zealand (NZ 3), and Italy (IT)). A statistically significant increase was seen in sites in Belgium, Italy and Spain between 2020-21 and 2021–22 (*p* < 0.05). There was also a statistically significant increase in the site in Italy between 2019-20 and 2021–22 (*p* < 0.05). In the New Zealand site where 3-MMC was found in both 2020–21 and 2021–22, there was also a statistically significant increase (*p* < 0.05). In most sites, there was an increase in mass loads over New Year's Eve and New Year's Day.

Eutylone was analysed over the two most recent campaigns. The three sites in New Zealand that were monitored over 2020–21 were also included in 2021–22. NZ 2 and NZ 3 showed similar trends for the different periods, with statistically significant increases found (*p* < 0.05), while eutylone at NZ 4 had no significant difference between the two sampling periods . Eutylone was also found at much lower levels in sites in Australia, North America, Brazil, Republic of Korea and specific sites in Europe (Fig. 2**B**). These differences for both 3-MMC and eutylone from 2020 to 21 to 2021–22 could reflect the impact of COVID-19 restrictions.

## 3 Discussion

The societal burden attributable to illicit drug use is increasing. For example, in Australia, the societal burden due to illicit drug use increased by 35% from 2003 to 2018 and was responsible for 72% of all burden due to poisonings and almost all burden related to drug use disorders (excluding alcohol) (Australian Institute of Health and Welfare 2021). However, the burden specifically related to NPS use is not easily quantified. This wastewater-based study provides a broader insight into NPS use and establishes which NPS are most used across sites in 16 countries and may potentially contribute to disease burden. Highest consumption was recorded for most NPS around the New Year period, indicating that consumption increases at festivals and parties. This may be due to higher consumption of regular consumers and/or “new” users who consume NPS intentionally or unintentionally. It must be noted that in countries where Lunar New Year is celebrated (i.e. China and the Republic of Korea), the December-January collection does not coincide with a specific holiday season. Nevertheless, this allows this work to also show the use of NPS in sites during a ‘normal’ time of the year.

### 3.1 Impact of the COVID-19 pandemic

The novelty of this work is emphasised by comparing the sampling campaigns covering sites before the COVID-19 pandemic (2019–20), when most severe COVID-19 related restrictions were in place (2020–21) and when most restrictions had eased (2021–22). Despite these three distinct periods and the impacts on the population, there was little difference in the total number of NPS detections. In fact, for a few compounds, high levels were seen during the 2020–21 period, such as eutylone in sites in New Zealand. Increases between 2020-21 and 2021–22 were observed in two sites in New Zealand, while a third had no significant change. These results are not unexpected, as pill testing over both periods carried out in New Zealand found that eutylone was the most commonly found cathinone (KnowYourStuffNZ 2023). Most compounds were at lower levels during the 2020–21 period particularly for 3-MMC in the European sites. It is acknowledged that the restriction of movement, as well as the cancellation of festivals and social gatherings likely resulted at these lower levels.

Moreover, the use of synthetic cathinones such as 3-MMC and eutylone is linked with that of MDMA (Pascoe et al., 2022). For example, the increase in eutylone in the summer of 2020–21 was hypothesised to be due to a reduction of MDMA in the country (Radio New Zealand 2022; Radio New Zealand 2021). MDMA was reported to have declined during 2020 in Europe, Australia and Canada, which could have resulted in the increased levels of 3-MMC and/or eutylone in this work (Bade et al., 2021; Been et al., 2021; Alygizakis et al., 2021; Statistics Canada 2023).

With the same time periods being monitored over the three years, this study is able to provide a ‘snapshot’ of NPS consumption. As such, the primary difference between the 2020–21 collection and those before and after is the COVID-19 pandemic. We therefore hypothesise that this was the driving factor for the differences observed.

### 3.2 Spatial and temporal trends

With samples collected at the same time every year, it is possible to see spatial and temporal changes in consumption trends (Supporting Information, Fig. S1). Many NPS seemingly follow regional trends. For example, mitragynine has highest loads in sites in the United States while eutylone and mephedrone were most common in New Zealand, 3-MMC and N-ethylhexedrone in Europe and 2F-DCK in China. Some NPS that were initially detected in specific regions spread to additional parts of the world in the third sampling campaign. For instance, 3-MMC was initially found in Europe, then spread to Oceania in 2020–21 and North America in 2021–22. Eutylone that was only detected in Oceania and North America in the second campaign but was detected in Europe in the last campaign, 2021–22. This study has identified the potential origin of NPS use around the world and could be used to monitor rapid changes in global consumers habits.

These results suggest that reducing NPS use and protecting public health should not only be done at the national level; it requires an organised global campaign. They also show the global spread of NPS in spite of current legislation. There are blanket bans on any NPS in countries such as China, New Zealand and Australia, while compound-specific bans also exist internationally. For example, the Convention on Psychotropic Substances 1971 from the United Nations is a treaty designed to control psychoactive drugs. The World Health Organisation (WHO) Expert Committee on Drug Dependence meets regularly to discuss the possible addition of new substances to the list of controlled drugs. In recent years, the NPS eutylone, N-ethylhexedrone and N-ethylpentylone have been added to the convention, while 3-MMC is currently under review and mitragynine is under surveillance (International Narcotics Control Board 2021; World Health Organization 2021; World Health Organization 2022).

#### 3.2.1 Synthetic cathinones

The synthetic cathinones are stimulants that constitute the largest group of NPS reported to the UNODC EWA (United Nations Office on Drugs and Crime 2021) and 3-MMC, eutylone, N-ethylhexedrone and mephedrone were all found in this work. 3-MMC is of particular concern in Europe. Since it was first reported in 2012 in Sweden, it has gained in popularity, particularly since 2020 (European Monitoring Centre for Drugs and Drug Addiction 2022). This is reflected in acute poisonings in the Netherlands (Nugteren-van Lonkhuyzen et al., 2022), while it was one of the most common NPS seized in Italy between May and October 2020 (Vincenti et al., 2021), and was also the NPS with the highest mass loads found in a recent national study conducted in Italy (Salgueiro-González et al., 2022). This European prevalence is reflected in Fig. 1. In the sites with multiple years of data, an increase in measured mass loads is evident. This increase in use has unfortunately resulted in at least 291 acute poisonings and 27 deaths in Europe (European Monitoring Centre for Drugs and Drug Addiction 2022). In early 2022, after a risk assessment report of 3-MMC commissioned by the EMCDDA, the European Commission recommended control measures and member states were given six months to introduce national legislation (European Monitoring Centre for Drugs and Drug Addiction 2022). The WHO has also decided to include 3-MMC amongst nine NPS for critical review by its Expert Committee on Drug Dependence because of its dependence-producing properties and potential harms (World Health Organization 2022).

In this study, the only sites outside of Europe where 3-MMC was found were in New Zealand and the United States, albeit at much lower levels than in Europe. There is no current literature around the use of 3-MMC in the United States or New Zealand but the New Zealand drug information website, High Alert, did release an article about 3-MMC in mid-2021 (HighAlert 2021). In New Zealand in late 2020, it was reported that there was also a decline in MDMA, which was maintained through 2021 (New Zealand Police 2021; New Zealand Police 2022). With some of the sites in the New Zealand locations incorporating music festivals, it is possible that the 3-MMC measured may have been from the consumption of adulterated MDMA. This hypothesis is supported by a study from the United Kingdom on drug use at festivals, which found that as MDMA detection decreased, cathinone detections increased and 3-MMC represented more than 20% of all cathinones found (Pascoe et al., 2022).

Mephedrone (4-methylmethcathinone) is an isomer of 3-MMC and was one of the most popular NPS in the early 2010s but has maintained its popularity despite legislation restrictions (Hutton, 2020). In the three years of this work, it has been found at relatively low levels, particularly in New Zealand and Australia. However, in 2021–22, it was also found in a site in Spain.

In this work, eutylone had highest levels in New Zealand. In late 2020, the New Zealand Drug Information website put out an alert for eutylone (HighAlert, 2021). Over the 2020–21 summer festival season in New Zealand, testing of party drugs across the country found that up to 50% of samples thought to be of MDMA contained eutylone. This reduced to around 10% the following year (Radio New Zealand 2022). However, in Fig. 2, similar levels were obtained across both years while site NZ 2 showed a statistically significant increase in 2021–22. In other sites where eutylone was monitored over 2020–21 and 2021–22, there was a decrease in sites in Australia and Canada, while eutylone was found for the first time in samples from sites in Italy, Belgium, and the Republic of Korea. According to seizure data, the most common NPS stimulants in Europe were 3-MMC and N-ethylhexedrone (European Monitoring Centre for Drugs and Drug Addiction. 2022), so it is interesting that some eutylone was found in several European sites. N-ethylhexedrone was only found in a couple of European sites (in Spain, Belgium and Sweden), with quite high levels found in a site Sweden.

#### 3.2.2 Mitragynine

Mitragynine is the primary alkaloid derived from a plant (kratom) found in South-East Asia, where it is traditionally used to combat fatigue and improve work productivity (Cinosi et al., 2015; Suwanlert, 1975). In recent years, it has become particularly popular in the United States as a ‘legal high’ for its stimulant and/or opioid-like effects (Tobacyk et al., 2022). The U.S. Food and Drug Administration has repeatedly warned of the dangers of kratom, including addiction, abuse and dependence (United States Food and Drug Administration 2022). It is not currently federally regulated in the United States, but some states have banned the substance, while others have imposed age restrictions. Use of the compound is legal in all the sites analysed in this study in the United States, but age restrictions may be in place to limit consumption by minors. Estimates of how many people use kratom in the United States vary, but the National Institute on Drug Abuse estimate that 0.6% (i.e. 1.7 million people) of the population aged 12 or older in 2021 reported using kratom in the past 12 months (Substance Abuse and Mental Health Services Administration 2022). Amongst the other sites in this project, mitragynine was only legal in Brazil, Belgium, Canada, Spain, and Greece. The legal status of mitragynine was not necessarily reflected in the measured mass loads calculated in this work. While sites in the United States had by far the highest levels of mitragynine, the next highest were in Sweden, Slovenia, and Australia – where it is illegal.

#### 3.2.3 2F-deschloroketamine

2F-deschloroketamine (2F-DCK) is an analogue of ketamine that has emerged over the past few years. Ketamine has been one of the drugs of most concern in China but major interventions have resulted in a large decline in registered ketamine users (United Nations Office on Drugs and Crime 2020). In recent years, ketamine analogues have emerged and become particularly prevalent in South-East Asia, including China. The highest levels of 2F-DCK were found in samples from one site in China and were similar to those previously reported (Shao et al., 2021; Li et al., 2022). It was also evident that there was no weekend (New Year) peak in use in the Chinese site, in contrast to other sites where 2F-DCK was found (e.g. Spain and Canada). It was also found infrequently in sites in the United States, Italy, and Iceland, at relatively low levels. According to the UNODC EWA, 2F-DCK is the second-most prevalent dissociative – behind ketamine – in all reporting countries since 2020 (United Nations Office on Drugs and Crime 2022).

#### 3.2.3 Designer benzodiazepines

Etizolam and clonazolam are designer benzodiazepines, a class associated with the greatest fatalities (United Nations Office on Drugs and Crime 2021) and hence of greatest public health concern. Etizolam was found in eight countries in this work, with most at or below our limit of quantification. However, a site in Iceland had the highest mass loads. Clonazolam was only found in this same site in Iceland. According to the Nordic Health and Welfare Statistics, Iceland has the highest prescription sales rate of benzodiazepines of all Nordic countries (Nordic Health and Welfare Statistics 2022). Both etizolam and clonazolam have been sold as counterfeit Xanax (Blakey et al., 2022) so it is possible that the high etizolam levels in Iceland were due to illicit or unwitting use of Xanax/alprazolam.

### 3.3 Future perspectives

The SPE method used for this work has previously been validated for synthetic cathinones, phenethylamines and opioids (Bade et al., 2020). However, recoveries for synthetic cannabinoids, benzodiazepines and plant-based NPS range between 10 and 50% (data not shown). Additionally, studies have shown that acidified conditions are unsuitable for the optimal quantification of cannabinoids and benzodiazepines (Bade et al., 2021; Pandopulos et al., 2020). While internal standards were included in this work to help cater for losses during extraction, it is possible that the limited detection frequency of the designer benzodiazepines and synthetic cannabinoids could be due to their instability in acidic conditions. To make a single method suitable for such a wide range of classes is challenging, but several methods have been developed to detect multiple classes of NPS in wastewater (Bade et al., 2020; O’Rourke and Subedi, 2020). As the variety and number of NPS continue to rise, it is important to continue developing new methods suitable to cover a wide number of classes in a single extraction.

The results of this study highlighted the need for a global campaign, as the issue of NPS use affects all countries to a different level and degree. Therefore, organisations, such as the UNODC and the EMCDDA, could adopt a similar wastewater analysis approach as a surveillance tool for NPS, since it allows the assessment of larger populations while minimising costs, provides data in an objective way, reducing the impact of self-reported data and presents no ethical implications, as the samples are anonymous. Annual and targeted (e.g., New Year period, music festivals and other special events) campaigns could act as a warning system for the prevalence of NPS.

## 4 Conclusion

Monitoring and surveillance of NPS internationally is an ongoing and complex problem. This wastewater-based study provides an insight into the NPS market internationally over the past three years, including before, during and after the COVID-19 pandemic. The use of NPS was mainly lower during the pandemic with a few exceptions, such as eutylone in specific sites. Higher NPS consumption around the world was found during the New Year holiday period and there were specific regional trends in which NPS was detected. For example, mitragynine had highest loads in sites in the United States, eutylone and mephedrone in New Zealand, 3-MMC and N-ethylhexedrone in Europe and 2F-DCK in China. These data indicate the promise of more systematic wastewater analyses to identify and monitor trends in the use of specific NPS in different populations and identify temporal and spatial patterns in their global spread of use.

## 5 Materials and methods

### 5.1 Compounds

A total of 52 compounds were analysed across the three years of this project, with between 26 and 34 analysed each year (Table S2).

### 5.2 Sampling campaigns

All information related to the wastewater collection and sites (collection dates, flow rates and population) can be found in the Supplemental Files (Table S3). As part of an ongoing global surveillance program, the number of countries (and sites) has increased from 8 (12) in the first iteration to 10 (25) and finally 16 (47). Data pertaining to the first two campaigns have been previously published (2019–20) (Bade et al., 2021) and 2020–21 (Bade et al., 2022). The population covered has increased from five to more than 17 million inhabitants. All samples were collected at the end of December – early January, coinciding with the New Year period. The first sampling campaign was performed before the COVID-19 pandemic, the second during the pandemic and the third when restrictions were ended/eased or presented only in a few countries.

### 5.3 Sampling, instrumentation and quality control

The best practice for WBE protocols were followed to minimise uncertainties relating to sample collection, storage and analytical methodology (Castiglioni et al., 2013). Briefly, 24-h composite influent wastewater was collected for between one and nine consecutive days. The wastewater was acidified upon collection and stored at -20 °C until sample treatment.

All samples were analysed using validated targeted LC-MS/MS methods (Bade et al., 2020; Bade et al., 2023). Procedural blanks were run throughout the analysis, after every 10 injections with the aim to identify any contamination originating from solvents and laboratory conditions. A quality control (blank spiked with 100 ng/L standards) was analysed after every 20 injections. Instruments were cleaned before the analysis according to vendors’ recommendations.

For quantification purposes, both transitions needed to be present, while the ion ratio (within 20%) as well as retention time had to compare with the standard (within 2%). If only one transition was present, the compound was deemed at above the limit of detection (LOD) but below the limit of quantification (LOQ). For calculation purposes, this was given as the midpoint between the LOD and LOQ. As no analyte-specific internal standards were used for this method, quantification was based on the peak area ratios between native and surrogate internal standards compared to an external calibration curve. All data were acquired and processed with SCIEX OS. Further details about the analytical methodology are provided in the Supplemental Files.

### 5.4 Calculations

For each compound, a calibration curve of up to 11 points was constructed from 0.1 to 10,000 ng/L. Concentrations were calculated using the isotope dilution method and processed using SCIEX OS or Multiquant 3.0.2. As labelled internal standards were not available for the NPS in this work, a surrogate internal standard was utilised (Table S4). The flow rates and population data provided by each collaborating laboratory (Table S3) were then used to calculate excreted mass loads (Table S4 for 2022–23; (2019–20 (Bade et al., 2021) and 2020–21 (Bade et al., 2022)).

### 5.5 Statistical analysis

Statistical tests were performed on all sites and compounds where more than one year of data was available. For sites with two years of data, independent t-tests were performed. For sites with three years of data, a one-way ANOVA was performed, followed by a pairwise *t*-test with Bonferroni correction. Differences were deemed statistically significant with *p* < 0.05. All statistical analyses were performed using R, version 4.2.1.

## CRediT authorship contribution statement

**Richard Bade:** Conceptualization, Methodology, Formal analysis, Investigation, Writing – original draft, Funding acquisition. **Nikolaos Rousis:** Investigation, Formal analysis, Writing – review & editing. **Sangeet Adhikari:** Resources, Writing – review & editing. **Christine Baduel:** Resources, Writing – review & editing. **Lubertus Bijlsma:** Resources, Writing – review & editing. **Erasmia Bizani:** Resources, Writing – review & editing. **Tim Boogaerts:** Resources, Writing – review & editing. **Daniel A. Burgard:** Resources, Writing – review & editing. **Sara Castiglioni:** Resources, Writing – review & editing. **Andrew Chappell:** Resources, Writing – review & editing. **Adrian Covaci:** Resources, Writing – review & editing. **Erin M. Driver:** Resources, Writing – review & editing. **Fernando Fabriz Sodre:** Resources, Writing – review & editing. **Despo Fatta-Kassinos:** Resources, Writing – review & editing. **Aikaterini Galani:** Resources, Writing – review & editing. **Cobus Gerber:** Resources, Supervision, Writing – review & editing. **Emma Gracia-Lor:** Resources, Writing – review & editing. **Elisa Gracia-Marín:** Resources, Writing – review & editing. **Rolf U. Halden:** Resources, Writing – review & editing. **Ester Heath:** Resources, Writing – review & editing. **Felix Hernandez:** Resources, Writing – review & editing. **Emma Jaunay:** Resources, Formal analysis, Writing – review & editing. **Foon Yin Lai:** Resources, Writing – review & editing. **Heon-Jun Lee:** Resources, Writing – review & editing. **Maria Laimou-Geraniou:** Resources, Writing – review & editing. **Jeong-Eun Oh:** Resources, Writing – review & editing. **Kristin Olafsdottir:** Resources, Writing – review & editing. **Kaitlyn Phung:** Resources, Writing – review & editing. **Marco Pineda Castro:** Resources, Writing – review & editing. **Magda Psichoudaki:** Resources, Writing – review & editing. **Xueting Shao:** Resources, Writing – review & editing. **Noelia Salgueiro-Gonzalez:** Resources, Writing – review & editing. **Rafael Silva Feitosa:** Resources, Writing – review & editing. **Cezar Silvino Gomes:** Resources, Writing – review & editing. **Bikram Subedi:** Resources, Writing – review & editing. **Arndís Sue Ching Löve:** Resources, Writing – review & editing. **Nikolaos Thomaidis:** Resources, Writing – review & editing. **Diana Tran:** Resources, Writing – review & editing. **Alexander van Nuijs:** Resources, Writing – review & editing. **Taja Verovšek:** Resources, Writing – review & editing. **Degao Wang:** Resources, Writing – review & editing. **Jason M. White:** Resources, Writing – review & editing. **Viviane Yargeau:** Resources, Writing – review & editing. **Ettore Zuccato:** Resources, Writing – review & editing. **Jochen F. Mueller:** Supervision, Resources, Funding acquisition, Writing – review & editing.

## Declaration of Competing Interest

RUH and EMD are cofounders of AquaVitas, LLC, Phoenix, Arizona, United States, an Arizona State University startup company providing commercial services in wastewater-based epidemiology. RUH also is the founder of OneWaterOneHealth, a nonprofit project of the Arizona State University Foundation.

## Data Availability

Data will be made available on request

## Appendix Supplementary materials

Image, application 1

Image, application 2
