# Supplementary material for: Three years of wastewater surveillance for new psychoactive substances from 16 countries
Source: Water Res X. 2023 Apr 6;19:100179. doi: 10.1016/j.wroa.2023.100179 (PMC10151418; doi:10.1016/j.wroa.2023.100179)
Supplement: Supplementary file 1 [file mmc1.pdf]

Supplemental Files

Three years of wastewater surveillance for new psychoactive substances in 16 countries

Richard Bade[a,*], Nikolaos Rousis[a], Sangeet Adhikari[b,c], Christine Baduel[d], Lubertus Bijlsma[e], Erasmia Bizani[f], Tim Boogaerts[g], Dan Burgard[h], Sara Castiglioni[i], Andrew Chappell[j], Adrian Covaci[g], Erin M. Driver[c,k], Fernando Fabriz Sodre[l], Despo Fatta-Kassinos[m], Felix Hernandez[e], Aikaterini Galani[f], Cobus Gerber[n], Emma Gracia-Lor[o], Elisa Gracia-Marín[e], Rolf U. Halden[b,c,k,p], Ester Heath[q], Emma Jaunay[n], Foon Yin Lai[r], Heon-Jun Lee[s], Maria Laimou-Geraniou[q], Jeong-Eun Oh[s], Kristin Olafsdottir[t], Kaitlyn Phung[j], Marco Pineda Castro[u], Magda Psichoudaki[m], Xueting Shao[v], Noelia Salgueiro-Gonzalez[i], Rafael Silva Feitosa[l], Cezar Silvino Gomes[w], Bikram Subedi[x], Arndís Sue Ching Löve[t], Nikolaos Thomaidis[f], Diana Tran[h], Alexander van Nuijs[g], Taja Verovšek[q], Degao Wang[v], Jason M. White[n], Viviane Yargeau[u], Ettore Zuccato[i], Jochen Mueller[a]

[a] Queensland Alliance for Environmental Health Sciences (QAEHS), The University of Queensland, 20 Cornwall Street, Woolloongabba, Queensland 4102, Australia

[b] School of Sustainable Engineering and Built Environment, Arizona State University, Tempe, AZ, 85281, USA

[c] Biodesign Center for Environmental Health Engineering, Biodesign Institute, Arizona State University, 1001 S. McAllister Ave., Tempe, AZ 85281, USA

[d] Université Grenoble Alpes, CNRS, IRD, Grenoble INP, IGE, Grenoble, France

[e] Environmental and Public Health Analytical Chemistry, Research Institute for Pesticides and Water, University Jaume I, Avda, Sos Baynat s/n, E-12071 Castellón, Spain

[f] Laboratory of Analytical Chemistry, Department of Chemistry, National and Kapodistrian University of Athens, Panepistimiopolis Zografou, 15771 Athens, Greece

[g] Toxicological Center, Department of Pharmaceutical Sciences, University of Antwerp, 2610 Wilrijk, Belgium

[h] Department of Chemistry and Biochemistry, University of Puget Sound, Tacoma, WA 98416, United States

[i] Istituto di Ricerche Farmacologiche Mario Negri IRCCS, Department of Environmental Health Sciences, Via Mario Negri 2, 20156, Milan, Italy

[j] Institute of Environmental Science and Research Limited (ESR), Christchurch Science Centre: 27 Creyke Road, Ilam, Christchurch 8041, New Zealand

[k] AquaVitas, LLC, Scottsdale, Arizona, 85251, United States

[l] Institute of Chemistry, University of Brasília, Brasília, DF, 70910-000, Brazil

[m] Nireas-International Water Research Centre and Department of Civil and Environmental Engineering, University of Cyprus, P.O. Box 20537, 1678, Nicosia, Cyprus

[n] Health and Biomedical Innovation, UniSA: Clinical and Health Sciences, University of South Australia, Adelaide 5001, South Australia, Australia

[o] Department of Analytical Chemistry, Faculty of Chemistry, Complutense University of Madrid, Avenida Complutense s/n, 28040 Madrid, Spain

[p] OneWaterOneHealth, Arizona State University Foundation, 1001 S. McAllister Avenue, Tempe, AZ 85287-8101, USA

[q] Jožef Stefan Institute and International Postgraduate School, Jamova 39, 1000 Ljubljana, Slovenia

[r] Department of Aquatic Sciences and Assessment, Swedish University of Agricultural Sciences (SLU), SE-75007 Uppsala, Sweden

[s] Department of Civil and Environmental Engineering, Pusan National University, Jangjeon-dong, Geumjeong-gu, Busan 46241, Republic of Korea

[t] University of Iceland, Department of Pharmacology and Toxicology, Hofsvallagata 53, 107 Reykjavik, Iceland

[u] Department of Chemical Engineering, McGill University, Montreal, QC, Canada

[v] College of Environmental Science and Engineering, Dalian Maritime University, No. 1 Linghai Road, Dalian, P. R. China, 116026

[w] Laboratory of Forensic Chemistry, Brazilian Federal Police, PB, Brazil

[x] Department of Chemistry, Murray State University, Murray, Kentucky 42071-3300, United States

*: Corresponding author: Richard Bade; email: r.bade@uq.edu.au

Analytical Methodology

Briefly, following filtration (GF/A 1.6 µm, Whatman, Kent, UK), the pH was adjusted to 4.5−5 using aqueous ammonia (28%) prior to solid phase extraction (UCT XtracT DAU, 500 mg/6 mL; UCT Inc., Bristol, PA, USA). The samples (100 mL) were loaded under gravity and the cartridges washed with a sodium acetate buffer (20 mM, 6 mL), acetic acid (0.1 M, 2 mL), and methanol (6 mL) before being air dried for 15 min. The dried cartridges were stored at −20 °C prior to shipping to The University of Queensland for analysis. On arrival at The University of Queensland, cartridges were again stored at −20 °C for no longer than 48 h before elution. A mixture of dichloromethane:isopropanol:aqueous ammonia (80:16:4 v/v/v, 6 mL) was used to elute the analytes from the cartridges and evaporated to approximately 200 µL under nitrogen at 40 °C. A solution of 1% HCl in methanol (20 µL) was then added, before being evaporated to dryness. The dry residue was reconstituted with 0.1% formic acid in methanol (20 µL) and 0.1% formic acid in ultrapure water (80 µL) to give a final volume of 100 µL and a concentration factor of 1000 times.

The site in Greece used a slightly different SPE method (Oasis MCX, 3cc, 60 mg). The cartridges were conditioned with methanol (3 mL) and acidified Milli-Q water (3 mL). Following filtration (GF/A 1.6 µm, Whatman, Kent, UK), the acidified wastewater samples (10 mL) were loaded under gravity. The cartridges were then washed with 40:60 acidified methanol: MilliQ water (acidified; 3 mL) and 20:80 acetonitrile:Milli-Q water. The cartridges were then dried for 30 mins under vacuum and shipped to The University of Queensland for analysis. On arrival at The University of Queensland, cartridges were again stored at −20 °C for no longer than 48 h before elution. The elution and analysis was carried out as in Shimko *et al* (1)

Instrumentation and Data Analysis (for 2019-20 and 2020-21) from Bade *et al* 2020 (2)

Analyses were performed using a Sciex ExionLC coupled to a Sciex 6500 + QTrap (Toronto, Canada), fitted with a TurboSpray IonDrive source. A Kinetex biphenyl column (150 × 2.1 mm × 1.7 µm) was used for the chromatographic separation at a flow rate of 0.3 mL/ min. The injection volume was 2 µL. The mobile phases used were 95% water with 5% methanol and 0.1% formic acid (solvent A) and 95% methanol with 5% water and 0.1% formic acid (solvent B). The initial percentage of B was 2% and increased to 20% over 2 min and 45% over the following 12 min. Over the next 5 min it was increased linearly to 100% and held for 0.1 min before being brought back to the initial percentage and kept steady for the final 2.9 min to equilibrate the system to give a total run time of 22 min. The ion source parameters were as follows: 450 °C; curtain gas, 20 psi; collision gas, medium; ion spray voltage, 5500 V; ion source gas 1 and ion source gas 2, 50 psi. Mass spectrometric analyses were performed in positive mode using multiple reaction monitoring (MRM). The two most abundant transitions of the precursor ion for each analyte and one for the deuterated internal standards were monitored. All data were acquired with Analyst 1.7 (Sciex) and processed using MultiQuant 3.0.2.

Instrumentation and Data Analysis (for 2021-22 samples) from Bade *et al* 2022 (3)

Samples were analysed using a Shimadzu Nexera LC-40 coupled to a SCIEX Triple Quad 7500 system. Chromatographic separation was achieved using a Phenomenex Kinetex Biphenyl (50 × 2.1 mm × 2.6 µm) column fitted with a SecurityGuard ULTRA Cartridges UHPLC Biphenyl 2.1 mm ID columns, at a flow rate of 0.35 mL/min and an injection volume of 2 µL. A mobile phase of 95:5 (v/v) MilliQ water: methanol with 0.1% formic acid (solvent A) and 95:5 (v/v) methanol: MilliQ water with 0.1% formic acid (solvent B) was used. The initial percentage of B was 5%, which was kept steady for the first 2 minutes. The concentration of B was linearly increased to 100% over 11 minutes and held for 2 minutes before being brought back to the starting conditions over 0.1 minutes and kept steady for the final 3.4 minutes to equilibrate the system. The total run time was 18.5 minutes. The mass spectrometer was run in scheduled multiple reaction monitoring (sMRM) mode in positive ion mode, with a 30 second retention time window around each analyte. In total, the mass spectrometric method time was approximately 15.5 minutes. The ion source gas 1 and 2 were set at 60 psi, curtain gas at 40 psi, ion source temperature at 450 °C and ion spray voltage at 2600 V.

For quantification purposes, both transitions needed to be present, while the ion ratio (within 20%) as well as retention time had to compare with the standard (within 2%). If only one transition was present, the compound was deemed at above the limit of detection (LOD) but below the limit of quantification (LOQ). For calculation purposes, this was given as the midpoint between the LOD and LOQ. As no analyte-specific internal standards were used for this method, quantification was based on the peak area ratios between native and surrogate internal standards compared to an external calibration curve. All data were acquired and processed with SCIEX OS.

Table S1: *Structures of Compounds found*

**Compound**	**Structure**
2F-Deschloroketamine (2F-DCK)	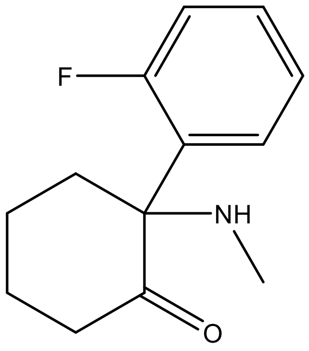
3-Methylmethcathinone (3-MMC)	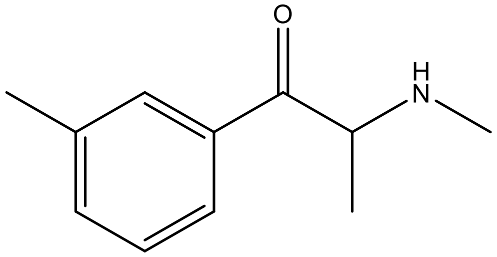
4-Fluoroamphetamine	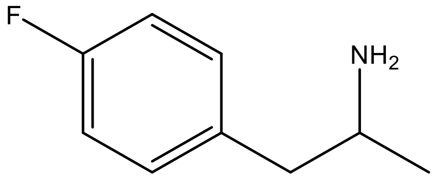
4-Methylethcathinone (4-MEC)	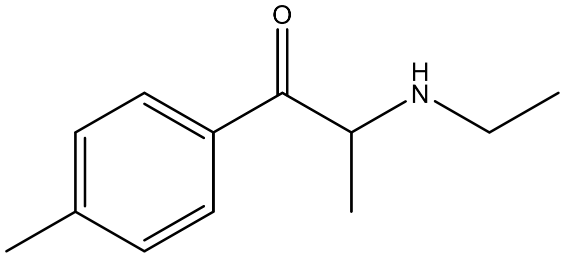
Clonazolam	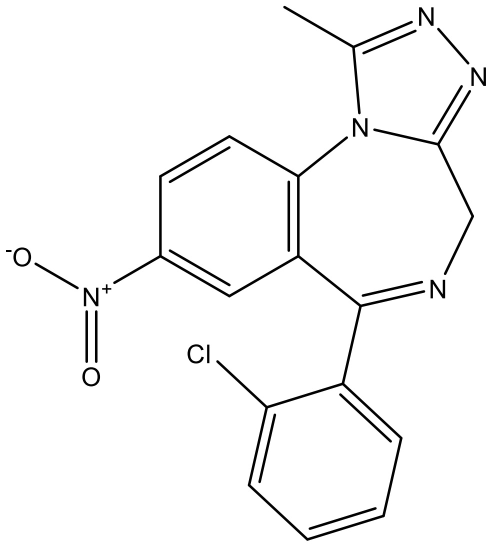

Etizolam	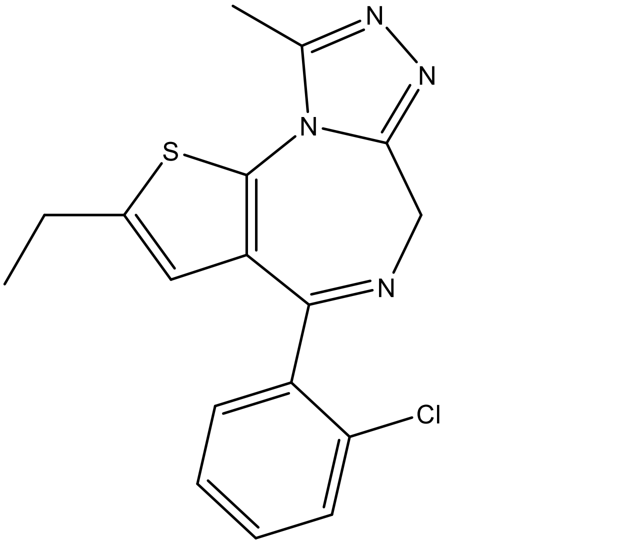
Ethylone	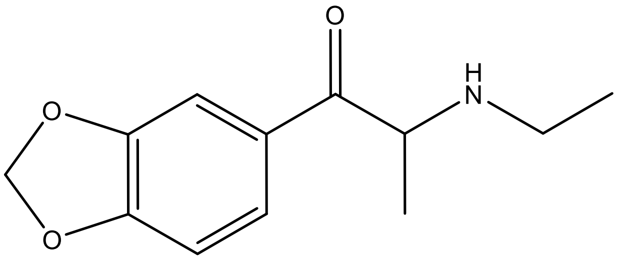
Eutylone	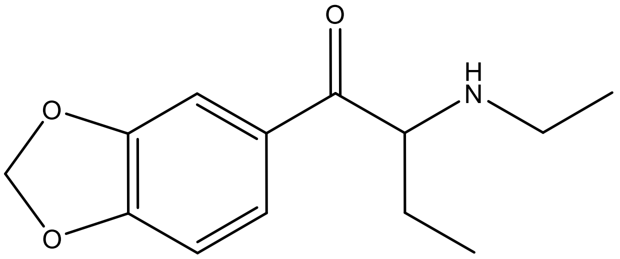
Methylenedioxypyrovalerone (MDPV)	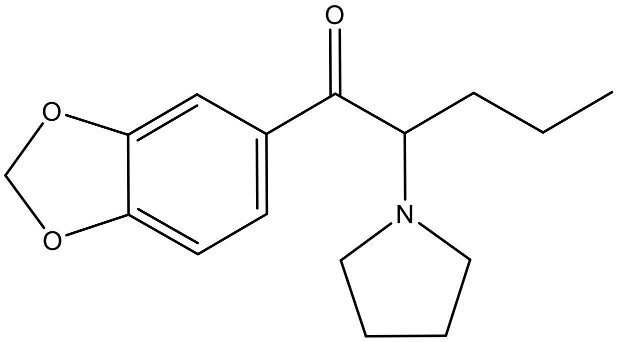
Mephedrone	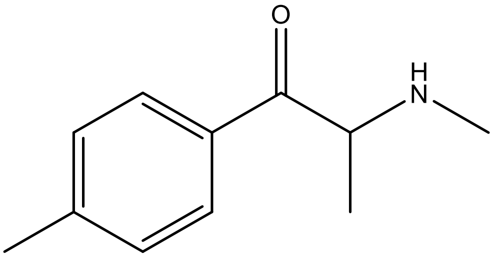

Methiopropamine	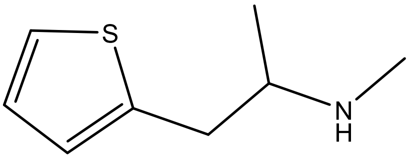
Methoxetamine	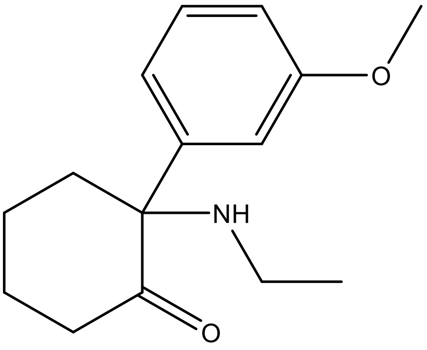
Methylone	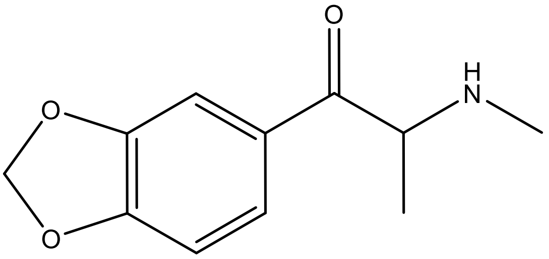
Mitragynine	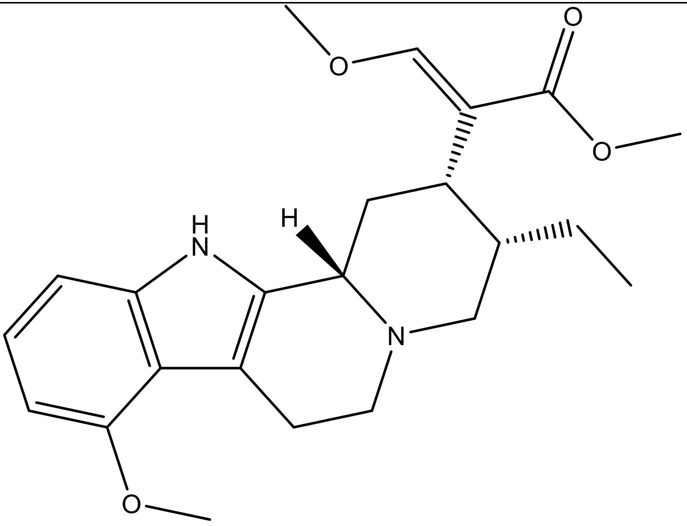
N-ethylhexedrone	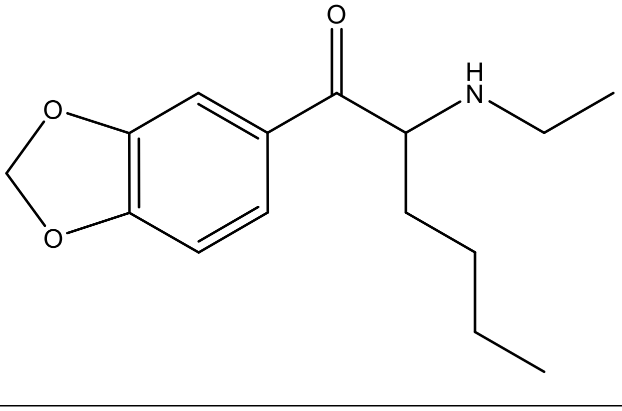

N-ethylpentylone	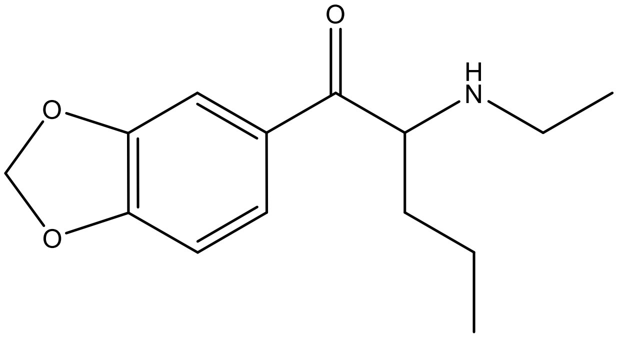
Pentylone	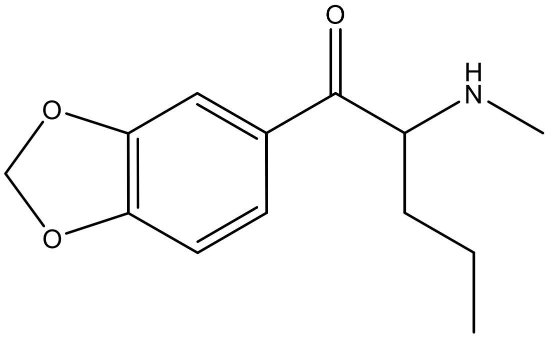
para-Methoxyamphetamine (PMA)	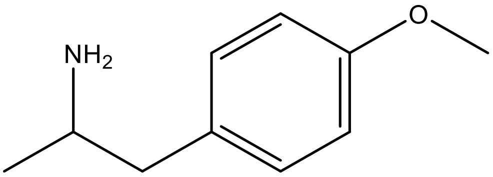

Figure S1: Regional trends in the use of NPS. Note: all sites within a country are aggregated so only a single box-and-whisker plot is shown for each country

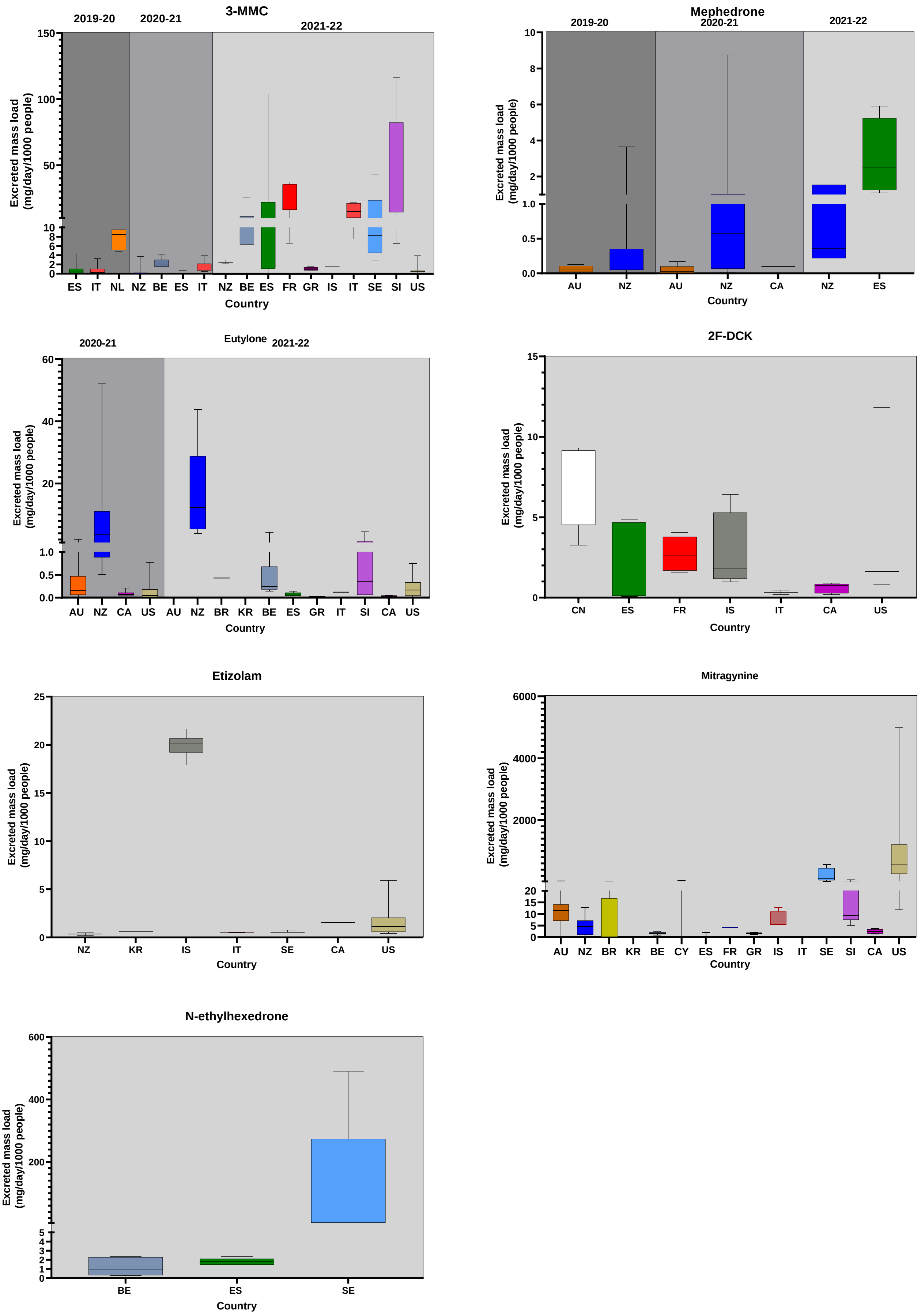

Table S2: Compounds targeted in each campaign

	Sampling campaign		
**Compound**	**2019-20**	**2020-21**	**2021-22**
25B-NBOMe	√	√	×
25C-NBOMe	√	√	√
25I-NBOMe	√	√	×
2F-Deschloroketamine	×	×	√
2-Methyl AP-237	×	×	√
2-Oxo-PCE	×	×	√
3-Ethylmethcathinone	√	√	×
3-Methylbuphedrone	√	√	×
3-Methylmethcathinone	√	√	√
4-Fluoroamphetamine	√	√	√
4-Fluoromethcathinone	√	√	×
4-Methylbuphedrone	√	√	×
4-Methylethcathinone	√	√	×
5F-EMB-PICA	×	×	√
5F-MDMB-PICA	×	×	√
5F-MDMB-PINACA	×	×	√
7-Hydroxymitragynine	×	×	√
AH-7921	√	√	×
AMB FUBINACA	×	×	√
AP-238	×	×	√
Brorphine	×	×	√
Buphedrone	√	√	×
Butylone	√	√	√
Butyryl fentanyl	√	√	×
Clonazolam	×	×	√
Cumyl pegaclone	×	×	√
Cumyl-5F-pegaclone	×	×	√
Dibutylone	×	×	√

Etizolam	×	×	√
Ethylone	√	√	×
Eutylone	×	√	√
Flualprazolam	×	×	√
Flubromazolam	×	×	√
Furanyl fentanyl	√	√	×
Isotonitazene	×	×	√
MDMB-4en-PINACA	×	×	√
MDPV	√	×	×
Mephedrone	√	√	√
Methcathinone	√	√	√
Methiopropamine	√	√	×
Methoxetamine	√	√	×
Methylone	√	√	√
Metonotazene	×	×	√
Mitragynine	×	×	√
N-Ethylheptedrone	×	×	√
N-Ethylhexedrone	×	×	√
N-Ethylpentylone	√	√	√
Pentylone	√	√	√
PMA	×	√	×
Protonitazene	×	×	√
U-47700	√	√	×
Valeryl fentanyl	√	√	×
Total analysed	26	27	34

Table S3: Flow Rates (in megalitres) and population of all sites in each campaign

	Flow rate (MegaLitres))													
	**AU1**	**AU2**	**AU3**	**AU4**	**BE**	**BR1**	**BR2**	**BR3**	**CA**	**CN**	**CY1**	**CY2**	**ES1**	**ES2**
Population	728,759	75,225	155,604	212,309	953,987	145,000	525,000	85,133	2,004,265	250,319[a]	31,079	205,212	178,141	1,370,216[a]
23-Dec-21	173.4	24.93	33.3	50.59										
24-Dec-21	175.2	25.4	35.2	53.46										
25-Dec-21	147.8	23.39	30.95	45.96										
26-Dec-21	143.7	23.51	31.17	46.62										
27-Dec-21	148.6	23.43	32.15	47.73										
28-Dec-21	154.2	23.11	32.11	47.87				43.01						
29-Dec-21	157.3	23.02	31.11	48.16	294.486	40.317	105.617	48.90	1573.603	68.326	8.803	25.53	31.868	285.10
30-Dec-21	157.6	22.91	30.86	47.92	271.159	53.735	122.143	41.24	1559.779	63.838	13.264	40.99	31.354	232.53
31-Dec-21	160.4	23.48	31.09	48.37	259.077	49.898	113.999	41.24	1581.552	65.505	6.493	45.72	35.24	262.66
1-Jan-22	140.4	22.62	30.2	45.89	554.537	37.707	101.823	30.75	1719.619	67.028	5.189	26.82	33.854	228.03
2-Jan-22	144.3	23.02	31.18	46.55	590.887	37.45	98.748	41.69	1619.914	63.517	5.204	25.9	32.564	213.83
3-Jan-22					406.129	43.623	111.559	39.16	1454.112	67.953	5.078	27.11	33.063	247.81
4-Jan-22					297.354	41.529	111.945	39.16	1527.466	66.397		26.65	32.811	277.13
5-Jan-22								36.32						
	**ES3**	**FR**	**GR**	**IS**	**IT**	**KR**	**NZ1**	**NZ2**	**NZ3**	**NZ4**	**SE1**	**SE2**	**SI1**	**SI2**
Population	87,484	471,326	3,568,758	107,000	1,022,389	922,656	28,736	12,000	39,500	37,000	878,800	200,000	270,305	129,000
23-Dec-21														
24-Dec-21														
25-Dec-21														
26-Dec-21														
27-Dec-21														
28-Dec-21					356.73				10		263.52	34.619	57.27	
29-Dec-21	32.694	482.4	856.9	145.4112	304.16	294.896		2	10	10	293.76	39.624	55.226	9.68
30-Dec-21	29.818	397.9	702.6	146.5344	303.58	289.69		2	10	10	268.704	39.683	54.028	24.024

31-Dec-21	27.858	345.6	727.6	143.1648	300.71	294.116	3	2	10	10	226.368	38.559	52.212	23.024
1-Jan-22	28.066	298.5	753.5	137.5488	307.29	279.805	3	2	10	10	308.448	46.887	45.85	19.528
2-Jan-22	29.269	283.2	638	146.1888	274.1	287.739	3	2	10	10	321.408	57.168	50.814	21.024
3-Jan-22	30.664	270.3	666.4	145.7568	284.28	290.104	3	2	10	10	345.6	55.443	55.274	21.12
4-Jan-22	35.574	306	688	135.4752		277.535		2		10				21.416
5-Jan-22														
	**SI3**	**SI4**	**US1**	**US2**	**US3**	**US4**	**US5**	**US6**	**US7**	**US8**	**US9**	**US10**	**US11**	**US12**
Population	51,667	25,141	893,000	19,456	16000	45,000	16,000	34,000	55,000	118,780	32,000	120,340	15,000	114,000
23-Dec-21														
24-Dec-21														
25-Dec-21														
26-Dec-21			394.8134											
27-Dec-21			368.0156										5.83	
28-Dec-21			359.9157	13.3			20.36			56.4				
29-Dec-21	8.91	4.408	342.9589	12.2	8.63							56.02		31.79
30-Dec-21	8.97	3.452	348.8256	13.0		27.43		16.29		55.05				
31-Dec-21	9.75	3.556	342.5804	19.4					51.85		19.81			
1-Jan-22	7.86	3.184	314.8363	60.4										
2-Jan-22	8.286	3.553	659.3092	34.2			16.05	17.48						30.28
3-Jan-22	8.11	3.419		21.6	9.63	22.19			51.85		22.18	45.84		
4-Jan-22		3.499				22.72	52.88	11.17			38.74		6.23	31.79
5-Jan-22					9.38					80.9		42.69	6.23	
6-Jan-22									51.97					
	**US13**	**US14**	**US15**	**US16**	**US17**									
Population	313800	413000	9400	12500	8700									
23-Dec-21														
24-Dec-21														
25-Dec-21														
26-Dec-21														

27-Dec-21														
28-Dec-21	51.78	51.03	0.42	2.64	1.94									
29-Dec-21	51.84	49.92	0.31	2.64	2.06									
30-Dec-21	51.8	50.08	0.33	2.64	2.09									
31-Dec-21														
1-Jan-22														
2-Jan-22														
3-Jan-22														
4-Jan-22	51.78	51.44	0.4	2.64										
5-Jan-22														
	**AU 1**	**AU 2**	**AU 3**	**AU 4**	**BE**	**CA**	**CN**	**ES**	**FJ1**	**FJ2**	**IT**	**KR**		
Population	728,759	75,225	155,604	212,309	953,987	1,942,044	213196[a]	177,088	45,000	180,000	821,604	922,656		
26-Dec-20											433.5			
27-Dec-20	155.1	22.9	31.6	46.7			65.62				307.9			
28-Dec-20	160.7	22.8	31.4	47.4	249.9	2185	59.74	36.9	9	36	327.2			
29-Dec-20	161.8	23	31.8	47.5	369.9	1918	62.2	36	9	36	481.4			
30-Dec-20	166.1	23	31.5	48.5	326	1826	67.34	37.87	9	36	440.7			
31-Dec-20	139.3	22.2	31.5	44.9	239.6	2153	66.89	34.81	9	36	431.2	282.1		
1-Jan-21	148.3	22.6	31.5	45.8	239.7	1755	65.79	44.21			820.8			
2-Jan-21	148.4	22.7	31.5	47.5	279.9	1784	68.36	31.36			833.8			
3-Jan-21	164.4	22.9		47.4	264.2	1840	67.32	41.2			383.7			
4-Jan-21								29.75						
	**NZ 1**	**NZ 2**	**NZ 3**	**US 1**	**US 2**	**US 3**	**US 4**	**US 5**	**US 6**	**US 7**	**US 8**	**US 9**	**US 10**	
Population	39,500	12,000	37,000	20,000	893,000	58,023	6,500	15,794	7,687	260,758	11,752	250	400,053	
26-Dec-20			8.98											
27-Dec-20	8.61	2.15	9.43	13.06	327.55	12.76	1.4	5.45	2.08	53.3	1.48	0.07	48.91	
28-Dec-20	10.25	2.51	10.56	13.17	351.17									
29-Dec-20	10.71	2.73	10.73	27.82	757.69									

30-Dec-20	11.32	2.52	11.43	35.02	538.82									
31-Dec-20	11.03	2.57	10.87	32.48	549.03	13.02	1.4	4.66	2.12	53.03	1.67	0.06	46.07	
1-Jan-21	14.26	2.43	10.07	21.39	946.21									
2-Jan-21	12.69	2.32	10.97	19.87	1047									
3-Jan-21	10.06	2.37	9.91		1162.2	13.74	1.4	5.41	2.42	55.83	1.55	0.06	49.82	
4-Jan-21			10.39											
	**AU 1**	**AU 2**	**AU 3**	**AU 4**	**CN**	**ES**	**IT**	**NL**	**NO**	**NZ 1**	**NZ 2**	**NZ 3**	**NZ 4**	**US**
Population*	728,759	75,225	212,309	155,604	228,439[a]	170,888	1,122,501	769,000	624,642[b]	120,000	19,283	28,736	20,000	893,000
25-Dec-19	139.4	22	46.5	38.2										313.1
26-Dec-19	138.7	22	47.3	30.9	71.4	31.8	286.1			53	1.779	2.6	2.1	301.8
27-Dec-19	151.7	27.8	47.9	30.7	72.7	32.5	327.9			54.4	2.004	2.9	2.3	294.2
28-Dec-19	149.5	21.8	47.7	30.6	71.9	32.5	284.8			54	2.175	3.1	2.5	278
29-Dec-19	138.9	21.7	45.9	30.6	71.9	31	310.7			51.5	2.212	3.3	2.6	274.8
30-Dec-19	153.8	22	47.9	30.8	72.2	31.6	306.5	157		53.8	2.244	3.4	2.7	279.7
31-Dec-19	158.9	22.3	49.5	30.6	71.6	30.5	338.7	162		52.8	2.468	3.8	2.6	328.7
1-Jan-20	158.9	21.4	44.8	29.7	69.8	35.1	286.5	146		48.2	2.549	3.9	2.7	323.9
2-Jan-20	158.9	21.9	47.4	30.5	70.8	31.1	317.6	155		51.4	2.384	3.5	2.6	467.6
3-Jan-20					70.6	31.6	308.5	186		57	2.316	3.4	2.5	
4-Jan-20								160						
6-Jan-20								160						

[a]: average population based on chemical parameters
[b]: With samples collected over multiple days, population and flow rates were based on the combined days

AU: Australia; BE: Belgium; BR: Brazil; CA: Canada; CN: China; CY: Cyprus; ES: Spain; FJ: Fiji; FR: France; GR: Greece; IS: Iceland; IT: Italy; KR: Republic of Korea; NL: the Netherlands; NZ: New Zealand; SE: Sweden; SI: Slovenia US: United States

References

1. Shimko KM, O'Brien JW, Barron L, Kayalar H, Mueller JF, Tscharke BJ, et al. A pilot wastewater-based epidemiology assessment of anabolic steroid use in Queensland, Australia. Drug Test Anal [Internet]. 2019 Jul 2;11(7):937–49. Available from: https://onlinelibrary.wiley.com/doi/10.1002/dta.2591

2. Bade R, Abdelaziz A, Nguyen L, Pandopulos AJAJ, White JMJM, Gerber C. Determination of 21 synthetic cathinones, phenethylamines, amphetamines and opioids in influent wastewater using liquid chromatography coupled to tandem mass spectrometry. Talanta [Internet]. 2020 Feb 1;208(August):120479. Available from: https://linkinghub.elsevier.com/retrieve/pii/S0039914019311129

3. Bade R, Eaglesham G, Shimko KM, Mueller J. Quantification of new psychoactive substances in Australian wastewater utilising direct injection liquid chromatography coupled to tandem mass spectrometry. Talanta [Internet]. 2023;251(July 2022):123767. Available from: https://doi.org/10.1016/j.talanta.2022.123767
